# Supplementary material for: Comparison of Transcriptomic Changes in Survivors of Exertional Heat Illness with Malignant Hyperthermia Susceptible Patients
Source: Int J Mol Sci. 2023 Nov 9;24(22):16124. doi: 10.3390/ijms242216124 (PMC10671540; doi:10.3390/ijms242216124)
Supplement: Supplementary file 1 [file ijms-24-16124-s001.zip › Supplemental Table S1.pdf]

SUPPLEMENTAL TABLE S1

	Passed HTT (n=10)	Failed HTT (n=10)
Age (years)	27 (6)	24 (7)
Height (m)	178 (5)	175 (8)
Body mass (kg)*	87.6 (14.3)	75.5 (9.6)
Body surface area (m^2)	2.09 (0.20)	1.91 (0.16)
Body fat (%)	19.8 (4.1)	17.1 (4.2)
Lean body mass (kg)	71.7 (10.4)	62.6 (8.7)
$\dot{V}O_{2max}$ ($L.min^{-1}$)	4.43 (0.82)	3.94 (0.48)
$\dot{V}O_{2max}$ ($ml.kg.min^{-1}$)	50.6 (5.6)	52.6 (6.2)

Supplemental Table S1. Mean (SD) subject characteristics and $\dot{V}O_{2max}$ data for the subjects recruited into the second phase: EHI subjects passing (n=10) and failing (n=10) the HTT. Descriptive data were produced for the subject characteristics and HTT data and the data were checked for normality. Comparisons of the data between the subjects passing and failing the HTT was made using independent T-tests (parametric data) and the Mann Whitney U (non-parametric data). Differences are reported for P<0.05. A statistically significant difference between groups (p <0.05) is denoted by an *.
