# Supplementary material for: Comparison of Transcriptomic Changes in Survivors of Exertional Heat Illness with Malignant Hyperthermia Susceptible Patients
Source: Int J Mol Sci. 2023 Nov 9;24(22):16124. doi: 10.3390/ijms242216124 (PMC10671540; doi:10.3390/ijms242216124)
Supplement: Supplementary file 1 [file ijms-24-16124-s001.zip › Supplemental Table S2.pdf]

SUPPLEMENTAL TABLE S2

		Phase 1 (0-30 min)	Phase 2	Phase 3
Clothing and equipment worn		Trousers, jacket, T.shirt, rucksack	Trousers, T.shirt	Trousers
Rectal (°C)	Pass	38.3 (0.4)	38.6 (0.3)*	38.7 (0.6)*
	Fail	38.6 (0.3)	39.0 (0.3)	39.4 (0.2)
Heart rate (beats.min^{-1})	Pass	171 (19)*	159 (21)*	159 (23)
	Fail	187 (9)	179 (11)	179 (12)
M_{sk} (°C)	Pass	35.8 (0.7)	34.6 (1.2)	34.7 (1.4)
	Fail	36.3 (0.8)	35.6 (1.4)	36.1 (1.7)
Rectal rate of rise (°C.hr^{-1})	Pass	1.8 (0.4)	1.4 (0.7)	0.3 (0.5)*
	Fail	2.3 (0.7)	1.8 (0.7)	1.1 (0.6)

Supplemental Table S2. Mean (SD) rectal and mean skin temperature and heart rate at the end of each phase and the rate of rise of heart rate for each group (n=10 in each group). A statistically significant difference between groups ($p < 0.05$) is denoted by an *.
