# Supplementary material for: Comparison of Transcriptomic Changes in Survivors of Exertional Heat Illness with Malignant Hyperthermia Susceptible Patients
Source: Int J Mol Sci. 2023 Nov 9;24(22):16124. doi: 10.3390/ijms242216124 (PMC10671540; doi:10.3390/ijms242216124)
Supplement: Supplementary file 1 [file ijms-24-16124-s001.zip › Supplemental Table S3 - MHS baseline ontology.pdf]

Comparison of transcriptomic changes in survivors of exertional heat illness with malignant hyperthermia susceptible patients

Supplemental Table S3: MHS baseline ontology

Upregulated MSigDB Hallmark

term	p-value	q-value	overlap_genes
UV Response Dn	0.000018	0.000518	[ABCC1, SFMBT1, ATRX, DYRK1A, ATP2B1, NR3C1, ADD3, SMAD7, SYNE1, TGFBR2, DLG1, SCAF8, KIT, TOGARAM1, CDK13]
TGF-beta Signaling	0.000021	0.000518	[SMURF2, APC, SMURF1, CTNNB1, ARID4B, MAP3K7, TGFBR1, SMAD7, HIPK2]
Mitotic Spindle	0.00191	0.031203	[DOCK4, ROCK1, SMC3, PCM1, CLIP1, DLG1, NIN, APC, PALLD, KIF5B, CNTRL, ARHGEF3, KIFAP3, ECT2]

Downregulated MSigDB Hallmark

term	p-value	q-value	overlap_genes
Oxidative Phosphorylation	1.20E-16	4.90E-15	[MRPS15, FH, NDUFB8, NDUFB7, MGST3, MRPS12, ATP5MC3, ETFB, PDHB, COX5B, HSD17B10, ATP5MC1, TIMM50, ATP5F1A, ATP5F1B, GRPEL1, CYC1, NDUFA8, NDUFA7, ATP5PD, NDUFA3, NDUFA1, NDUFC2, COX6B1, OXA1L, NDUFS8, NDUFS7, NDUFAB1, VDAC2, SLC25A5]
Myc Targets V1	1.16E-07	2.37E-06	[CCT3, NOP56, EIF4A1, RANBP1, KARS1, PHB, ILF2, RRP9, NME1, PSMD8, CDK4, APEX1, IMPDH2, NDUFAB1, POLD2, RPL14, NHP2, CYC1, SSBP1]
Myc Targets V2	4.01E-05	5.49E-04	[NOP56, EXOSC5, CDK4, PUS1, WDR74, IMP4, PHB, RRP9]
DNA Repair	5.35E-04	5.49E-03	[NT5C, GTF2A2, DAD1, CETN2, ERCC1, IMPDH2, POLR1C, POLR2G, ADA, TARBP2, NME1]
Unfolded Protein Response	9.41E-04	7.72E-03	[NOP56, EIF4A1, EXOSC5, SPCS1, NHP2, BANF1, EXOSC1, YIF1A, RRP9]
Adipogenesis	1.77E-03	1.21E-02	[SAMM50, NDUFB7, GRPEL1, MGST3, NDUFAB1, UBC, PEMT, CHCHD10, ABCB8, CYC1, REEP6, ETFB]
Fatty Acid Metabolism	2.84E-03	1.66E-02	[FH, LGALS1, UROD, NTHL1, APEX1, ERP29, REEP6, PDHB, HSD17B10, S100A10]

Upregulated GO Biological Process

term	p-value	q-value	overlap_genes
regulation of transcription by RNA polymerase II (GO:0006357)	1.29E-10	3.84E-07	[MAML1, ZNF292, JMJD1C, ARID4A, ARID4B, PRDM1, NR3C1, BACH1, ELK3, NR3C2, ZSCAN30, CREB3L2, EPC2, RNF111, ZNF281, ZBTB38, MAF, PRKAR1A, KAT6B, HOXB3, ZNF674, ZSCAN29, ZNF395, KMT2A, KMT2C, TGFA, BAZ2B, HTATSF1, HIF1A, PRDM10, RLF, RAD21, ZNF669, ZNF304, SUDS3, ZKSCAN4, RREB1, ZKSCAN1, ZBED4, BPTF, JAG1, AUTS2, SMURF2, PTCH1, SMARCA5, FLI1, MED13L, SMAD7, SETX, DLG1, FUBP3, SP3, CDK13, PHF2, DDX3X, MIDEAS, ZNF45, BRCA1, CHD2, CHD1, ZNF808, NKRF, ZNF644, LRRFIP1, NCOA2, PPP1R12A, ATRX, TET2, VEZF1, BAZ1A, ETV3, ARID1B, PHF20L1, MED23, KAT2B, PLSCR1, ELF1, ELF2, TFDP2, SLTM, TET3, KIT, CRY1, NCOA7, ZNF236, PPARA, RNASEL, KDM3A, TMF1, NR2C2, ATAD2B, ZNF507, ZNF624, E2F3, ZNF468, FNIP2, ZNF585A, ZNF189, NFYA, MGA, ...]
regulation of transcription, DNA-templated (GO:0006355)	3.65E-10	5.42E-07	[ZNF292, JMJD1C, ARID4A, ARID4B, PRDM1, NR3C1, BACH1, ELK3, NR3C2, ZSCAN30, CREB3L2, EPC2, RNF111, TRIM22, SFMBT1, ZNF281, ZBTB38, F2R, DYRK1A, MAF, PRKAR1A, KAT6B, HOXB3, ZNF674, ZSCAN29, KMT2E, ZNF395, KMT2A, TGFA, BAZ2B, HTATSF1, HIF1A, RLF, TAF5L, SBNO1, RAD21, ZNF669, ZNF304, SUDS3, ZKSCAN4, RREB1, ZKSCAN1, BPTF, CDK17, SMURF2, SMARCA5, ZBTB11, FLI1, MED13L, SMAD7, FUBP3, SP3, LIN54, SETD5, PHF2, SPIN4, MIDEAS, HP1BP3, ZNF45, BRCA1, CHD2, CHD1, ZNF808, NKRF, ZNF280C, ZNF644, LRRFIP1, NCOA2, USP47, ATRX, VEZF1, BAZ1A, ACTR8, ETV3, ARID1B, TGFBR1, PHF20L1, MED23, ELF1, ELF2, TFDP2, SLTM, KIT, CRY1, NCOA7, ZNF236, PPARA, PLCB1, ZMYND11, L3MBTL4, TRIM56, KDM3A, CIPC, RNF6, SPTY2D1, NR2C2, ATXN1L, NSD2, ZNF624, E2F3, ...]
positive regulation of transcription, DNA-templated (GO:0045893)	1.81E-07	1.79E-04	[DDX3X, MAML1, BRCA1, NR3C1, ELK3, NKRF, CREB3L2, RNF111, NCOA2, PPP1R12A, ZNF281, ZBTB38, F2R, ATRX, TET2, DYRK1A, VEZF1, ARID1B, TGFBR1, KAT2B, PLSCR1, ELF1, ELF2, TFDP2, KAT6B, TET3, HOXB3, NCOA7, PPARA, PLCB1, TRIM56, KMT2E, ZNF395, RNASEL, KDM3A, TMF1, KMT2A, KMT2C, RNF6, NR2C2, HIF1A, PRDM10, RLF, ATAD2B, SCAF8, ZNF507, ZNF304, E2F3, RREB1, ZBED4, BPTF, JAG1, NFYA, AUTS2, SMARCA5, CDC5L, FLI1, HIPK2, SMAD7, SETX, FUBP3, SP3, ASXL2, F2RL1, CTNNB1, CRLF3]
positive regulation of transcription by RNA polymerase II (GO:0045944)	8.37E-07	6.21E-04	[DDX3X, MAML1, BRCA1, NR3C1, ELK3, NKRF, RNF111, NCOA2, PPP1R12A, ZBTB38, ATRX, TET2, VEZF1, KAT2B, PLSCR1, ELF1, TFDP2, KAT6B, TET3, HOXB3, NCOA7, PPARA, ZNF395, RNASEL, TMF1, PAXIP1, KMT2A, KMT2C, NR2C2, HIF1A, PRDM10, RLF, ATAD2B, ZNF507, ZNF304, E2F3, RREB1, ZBED4, BPTF, JAG1, AUTS2, SMARCA5, CDC5L, HIPK2, SMAD7, SETX, FUBP3, SP3, ASXL2, F2RL1, CTNNB1, CDK13, CRLF3]
regulation of gene expression (GO:0010468)	1.63E-06	9.69E-04	[LIN54, MYOM1, DDX3X, SPIN4, HP1BP3, JMJD1C, BRCA1, PRDM1, NR3C1, BACH1, SLC8A1, AFF1, ZNF280C, ADNP2, DIP2A, TRIM22, KDM6A, NCOA2, MBNL2, FBXW7, ZNF281, ZBTB38, ATRX, ADAM10, ACTR8, TGFBR1, MED23, PLSCR1, KAT6B, ADNP, ZNF395, ROCK1, RNF6, SPTY2D1, NR2C2, TOB1, HIF1A, PRDM10, TAF5L, SBNO1, ZNF507, ATXN1L, NSD2, UBR5, GPBP1L1, RREB1, ANGPT1, NFYA, TDRD7, ZBTB11, PUM1, TNRC6C, PTPRC, FUBP3, SP3, ZNF217, TNRC6A, TNRC6B, TAF1]
positive regulation of histone H3-K4 methylation (GO:0051571)	2.21E-06	1.09E-03	[KMT2E, PAXIP1, AUTS2, KMT2A, CTNNB1, BRCA1]
regulation of nucleic acid-templated transcription (GO:1903506)	2.91E-06	1.12E-03	[LIN54, ZNF395, SPIN4, HP1BP3, JMJD1C, RNF6, SPTY2D1, NR3C1, NR2C2, BACH1, HIF1A, TAF5L, SBNO1, ZNF280C, NSD2, ATXN1L, GPBP1L1, RREB1, TRIM22, NCOA2, NFYA, ZNF281, ZBTB38, ATRX, ZBTB11, ACTR8, TGFBR1, MED23, KAT6B, SP3, ZNF217]
peptidyl-threonine modification (GO:0018210)	3.01E-06	1.12E-03	[ROCK1, GALNT3, PDPK1, DYRK1A, LMTK2, HIPK1, TGFBR1, TGFBR2, HIPK2, CDK5R1, TAF1]
protein polyubiquitination (GO:0000209)	4.81E-06	1.59E-03	[TNFAIP3, RNF6, BRCA1, HECTD1, UBR5, RFFL, TRIM22, SHPRH, ANKIB1, AREL1, SMURF2, FBXW7, SMURF1, SIAH1, FBXW2, KLHL20, RNF144A, RNF168, KBTBD6, PSME4, PELI1, CTNNB1, KBTBD7, TRIM56, TAF1]
protein modification by small protein removal (GO:0070646)	5.43E-06	1.61E-03	[USP47, USP25, USP38, SMURF2, USP42, CCP110, TNFAIP3, STAM, BRCA1, ACTR8, HIF1A, TGFBR1, SMAD7, SENP6, CYLD, KAT2B, APC, ATXN7, PSME4, ASXL2, SUDS3, USP30, MAP3K7]

Downregulated GO Biological Process

term	p-value	q-value	overlap_genes
mitochondrial electron transport, NADH to ubiquinone (GO:0006120)	1.28E-12	1.61E-09	[NDUFB9, NDUFA8, NDUFA13, NDUFB8, NDUFA7, NDUFB7, NDUFA12, NDUFA3, NDUFA1, NDUFC2, NDUFS8, NDUFS7, NDUFAB1]

aerobic electron transport chain (GO:0019646)	2.20E-12	1.61E-09	[NDUFB9, NDUFA8, NDUFA13, NDUFB8, NDUFA7, NDUFB7, NDUFA12, NDUFA3, NDUFA1, NDUFC2, COX5B, COX6B1, NDUFS8, NDUFS7, NDUFAB1, CYC1]
mitochondrial ATP synthesis coupled electron transport (GO:0042775)	2.78E-12	1.61E-09	[NDUFB9, NDUFA8, NDUFA13, NDUFB8, NDUFA7, NDUFB7, NDUFA12, NDUFA3, NDUFA1, NDUFC2, COX5B, COX6B1, NDUFS8, NDUFS7, NDUFAB1, CYC1]
mitochondrial respiratory chain complex I assembly (GO:0032981)	2.40E-11	8.33E-09	[NDUFB9, NDUFA8, NDUFA13, NDUFB8, NDUFA7, NDUFB7, NDUFA12, NDUFA3, NDUFA1, NDUFC2, OXA1L, NDUFS8, NDUFS7, NDUFAB1]
NADH dehydrogenase complex assembly (GO:0010257)	2.40E-11	8.33E-09	[NDUFB9, NDUFA8, NDUFA13, NDUFB8, NDUFA7, NDUFB7, NDUFA12, NDUFA3, NDUFA1, NDUFC2, OXA1L, NDUFS8, NDUFS7, NDUFAB1]
mitochondrial translational elongation (GO:0070125)	1.06E-10	2.64E-08	[MRPS15, MRPS27, MRPS24, MRPS12, MRPS18A, MRPS2, MRPL12, MRPL24, MRPS5, MRPL4, MRPL41, MRPL52, OXA1L, MRPL2, MRPS9, MRPL51]
mitochondrial translational termination (GO:0070126)	1.06E-10	2.64E-08	[MRPS15, MRPS27, MRPS24, MRPS12, MRPS18A, MRPS2, MRPL12, MRPL24, MRPS5, MRPL4, MRPL41, MRPL52, OXA1L, MRPL2, MRPS9, MRPL51]
mitochondrial translation (GO:0032543)	1.56E-10	3.38E-08	[MRPS15, NDUFA7, MRPS27, MRPS24, MRPS12, MRPS18A, MRPS2, MRPL12, MRPL24, MRPS5, MRPL4, MRPL41, MRPL52, OXA1L, MRPL2, MRPS9, MRPL51]
translational termination (GO:0006415)	3.47E-10	6.68E-08	[MRPS15, MRPS27, MRPS24, MRPS12, MRPS18A, MRPS2, MRPL12, MRPL24, MRPS5, MRPL4, MRPL41, MRPL52, OXA1L, MRPL2, MRPS9, MRPL51]
translational elongation (GO:0006414)	1.18E-09	1.96E-07	[MRPS15, MRPS27, MRPS24, MRPS12, MRPS18A, MRPS2, MRPL12, MRPL24, MRPS5, MRPL4, MRPL41, MRPL52, OXA1L, MRPL2, MRPS9, MRPL51]

Upregulated GO Molecular Function

term	p-value	q-value	overlap_genes
ubiquitin-protein transferase activity (GO:0004842)	0.00001	0.004794	[UBR3, TNFAIP3, RNF6, BRCA1, HECTD1, UBR5, RFFL, RNF111, TRIM22, SHPRH, ANKIB1, AREL1, SMURF2, SMURF1, SIAH1, MEX3C, FBXW2, PJA2, KLHL20, RNF168, RNF144A, RNF145, RFWD3, PELI1, MKRN2, BIRC6, TRIM56, TAF1]
methylated histone binding (GO:0035064)	0.000023	0.005337	[KMT2E, PHF2, SPIN4, ZMYND8, ATRX, CDYL, ZMYND11, CHD1, BPTF, TAF1]
methylation-dependent protein binding (GO:0140034)	0.000075	0.009229	[KMT2E, PHF2, SPIN4, ZMYND8, ATRX, CDYL, ZMYND11, CHD1, BPTF]
ubiquitin protein ligase activity (GO:0061630)	0.00008	0.009229	[SHPRH, ANKIB1, AREL1, SMURF2, SMURF1, SIAH1, UBR3, RNF6, MEX3C, RNF144A, RNF145, HECTD1, RFWD3, UBR5, PELI1, MKRN2, RFFL, RNF111, TRIM22, TRIM56]
ubiquitin-like protein ligase activity (GO:0061659)	0.000115	0.010574	[SHPRH, ANKIB1, AREL1, SMURF2, SMURF1, SIAH1, UBR3, RNF6, MEX3C, RNF144A, RNF145, HECTD1, RFWD3, UBR5, PELI1, MKRN2, RFFL, RNF111, TRIM22, TRIM56]
histone methyltransferase activity (H3-K36 specific) (GO:0046975)	0.00019	0.012679	[SETD5, SETD2, NSD2, ASH1L]
phosphatidylinositol-3,5-bisphosphate 3-phosphatase activity (GO:0052629)	0.000226	0.012679	[MTMR1, MTMR4, MTM1]
acetylation-dependent protein binding (GO:0140033)	0.000268	0.012679	[KMT2A, ZMYND8, PSME4, ATAD2B, TAF1]
lysine-acetylated histone binding (GO:0070577)	0.000268	0.012679	[KMT2A, ZMYND8, PSME4, ATAD2B, TAF1]
histone-lysine N-methyltransferase activity (GO:0018024)	0.000275	0.012679	[KMT2E, SETD5, SETD2, KMT2A, KMT2C, NSD2, ASH1L]

Downregulated GO Molecular Function

term	p-value	q-value	overlap_genes
oxidoreduction-driven active transmembrane transporter activity (GO:0015453)	1.54E-12	5.01E-10	[NDUFB9, NDUFA8, NDUFA13, NDUFB8, NDUFA7, NDUFB7, NDUFA12, NDUFA3, NDUFA1, NDUFC2, COX5B, CYB561D2, COX6B1, NDUFS8, NDUFS7]
NADH dehydrogenase (quinone) activity (GO:0050136)	6.50E-12	7.04E-10	[NDUFB9, NDUFA8, NDUFA13, NDUFB8, NDUFA7, NDUFB7, NDUFS8, NDUFS7, NDUFA12, NDUFA3, NDUFA1, NDUFC2]
NADH dehydrogenase (ubiquinone) activity (GO:0008137)	6.50E-12	7.04E-10	[NDUFB9, NDUFA8, NDUFA13, NDUFB8, NDUFA7, NDUFB7, NDUFS8, NDUFS7, NDUFA12, NDUFA3, NDUFA1, NDUFC2]
RNA binding (GO:0003723)	3.60E-06	2.92E-04	[POP5, EIF4A1, MRPS15, RTCB, MRPS12, GPATCH4, PARK7, RRP9, MRPL41, MRPL4, RBM4, MRPL2, LGALS1, RPL36, ARL6IP4, SEC61B, BUD23, UTP14A, RPP38, EIF5A, CCT3, MRPS27, MRPS24, PUS1, NOSIP, IMP4, ILF2, MRPS5, NME1, MRPS9, NHP2, RPP21, SNRPC, SLC25A5, RPP25, PPIB, KARS1, TRMT2A, MRPL12, HSD17B10, ATP5F1A, UBC, RPL14, GAR1, POLR2G, EXOSC1, NOP56, TRAP1, CPSF4, TRMT1, ELP5, NPM3, TARBP2, APEX1, EIF3G, NOL10, SSBP1, FAM50A]
ribonuclease P RNA binding (GO:0033204)	4.53E-05	2.94E-03	[POP5, RPP21, RPP25, RPP38]
ribonuclease P activity (GO:0004526)	6.99E-05	3.78E-03	[POP5, RPP21, RPP25, RPP38]
tRNA-specific ribonuclease activity (GO:0004549)	2.01E-04	9.33E-03	[POP5, RPP21, RPP25, RPP38]
endoribonuclease activity, producing 5'-phosphomonoesters (GO:0016891)	3.40E-04	1.29E-02	[POP5, APEX1, RPP21, RPP25, RPP38]
NADH dehydrogenase activity (GO:0003954)	3.56E-04	1.29E-02	[NDUFA13, NDUFS8, NDUFS7]
snoRNA binding (GO:0030515)	4.75E-04	1.54E-02	[NOP56, GAR1, NHP2, IMP4, RRP9]

Upregulated GO Cellular Component

term	p-value	q-value	overlap_genes
intracellular membrane-bounded organelle (GO:0043231)	6.13E-14	1.53E-11	[ITSN2, MAML1, ZMYND8, ZNF292, LDLRAD4, BACH1, GPATCH8, ELK3, KIAA1109, CNST, CREB3L2, ADNP2, PWWP2A, DIP2B, CCDC93, RNF111, TRIM22, TSPYL1, SFMBT1, ZNF281, INPP4A, PSME4, AKAP9, HOXB3, ZNF395, PAXIP1, AGAP1, HTATSF1, HIF1A, PRDM10, TP53BP2, PIP5K1A, RREB1, ZBED4, CDK17, ABCA1, SIAH1, EAF1, SMARCA5, STAM, FLI1, WAPL, SETX, FUBP3, CDK13, CPEB4, LIN54, SETD5, PHF2, SETD2, PRKAG2, ZNF45, BRCA1, OSBPL11, CLINT1, ZNF808, NKRF, REV3L, APPL2, UPF2, NCOA2, BRD1, TET2, ASH1L, RNF168, RNF144A, KAT2B, UGDH, PLSCR1, SLTM, TET3, SDE2, NCOA7, ZNF236, PPARA, PLCB1, PHLPP2, CIPC, TMEM165, SCAF8, FAM172A, NSD2, GPBP1L1, ZNF468, SF3B1, EPS15, NKTR, ZNF585A, CASP8AP2, NFYA, ERBIN, HIPK1, HIPK2, KBTBD6, PER3, RFWD3, ZNF217, CTNNB1, CRLF3, CDK5R1, ...]
nucleus (GO:0005634)	1.12E-09	1.40E-07	[MAML1, ZMYND8, ZNF292, JADE1, ARID4A, CRAMP1, ARID4B, PRDM1, NR3C1, BACH1, GPATCH8, ELK3, KIAA1109, AMMECR1L, CREB3L2, ADNP2, PWWP2A, DIP2B, MAP3K7, RNF111, TRIM22, TSPYL1, AGTPBP1, FBXW7, SFMBT1, ZNF281, ZBTB38, DYRK1A, ADAM10, CDYL, INPP4A, CLIP1, MAF, KAT6B, PSME4, WDFY1, HOXB3, ADNP, KMT2E, ZNF395, PAXIP1, KMT2A, KMT2C, ANKRD11, AGAP1, PDS5B, HTATSF1, STK4, HIF1A, PRDM10, GTF2E1, RLF, TAF5L, TOR1AIP1, ZFP36L2, SBNO1, ATXN7, UBR5, TP53BP2, PIP5K1A, ZNF304, SUDS3, ECT2, RREB1, ZBED4, BPTF, CDK17, SMURF2, SIAH1, SMARCA5, NUP153, FLI1, WAPL, SMAD7, SETX, PTPRE, CNOT6, DLG1, FUBP3, SP3, CDK13, CPEB4, RBM27, LIN54, SETD5, PHF2, SETD2, DDX3X, USP38, MCM9, HP1BP3, PRKAG2, TNFAIP3, CGAS, ZNF45, BRCA1, CHD2, CHD1, SYNE2, SYNE1, ...]
Golgi cis cisterna (GO:0000137)	3.12E-07	2.59E-05	[GOLGA8R, GOLGA8B, GOLGA8T, XYLT1, GOLGA8J, GOLGA8O, GOLGA8N, GOLGA8Q]
cis-Golgi network (GO:0005801)	4.57E-06	2.84E-04	[GOLGA8R, GOLGA8B, GOLGA8T, AKAP9, GOSR1, GOLIM4, GOLGA8J, GOLGA8O, GOLGA8N, GOLGA8Q]
Golgi cisterna membrane (GO:0032580)	6.40E-06	3.19E-04	[GOLGA8R, GOLGA8B, GOLGA8T, GOLGA8J, GOLGA8O, GOLGA8N, GOLGA8Q]
Golgi cisterna (GO:0031985)	1.81E-05	7.50E-04	[GOLGA8R, GOLGA8B, GOLGA8T, GOSR1, XYLT1, GOLGA8J, GOLGA8O, GOLGA8N, GOLGA8Q]
ISWI-type complex (GO:0031010)	1.24E-04	4.40E-03	[HMGXB4, SMARCA5, BAZ1A, BPTF]
NURF complex (GO:0016589)	2.26E-04	7.05E-03	[HMGXB4, SMARCA5, BPTF]
spindle (GO:0005819)	4.57E-04	1.26E-02	[PHLPP2, BOD1L1, TTL, CDC14A, WAPL, CYLD, NIN, TFDP2, KIF16B, CSPP1, TOGARAM1, CEP170, KIFAP3, ECT2, CLASP2]

membrane raft (GO:0045121)	8.86E-04	2.20E-02	[ABCA1, ITGB1, CR1, ANGPT1, ERLIN2, LAX1, TGFBR1, TGFBR2, PLSCR1, PIKFYVE, PTPRC, PRKAR1A, PRKAR2A]

Downregulated GO Cellular Component

term	p-value	q-value	overlap_genes
mitochondrial membrane (GO:0031966)	2.56E-20	3.71E-18	[NDUFA13, MRPS15, NDUFA12, MRPS12, MICOS13, MRPL41, MRPL4, MRPL2, MRPS27, MRPS24, TIMM23, NDUFC2, MRPS18A, MRPS2, MRPS5, COX6B1, MRPL52, OXA1L, MRPS9, MRPL51, HAX1, SLC25A19, VDAC2, SLC25A5, NDUFB9, NDUFB8, NDUFB7, TOMM40, ATP5MC3, ABCB8, PHB, PPOX, MRPL12, COX5B, MTLN, TIMM50, ATP5MC1, ATP5F1A, SAMM50, ATP5F1B, UBC, CYC1, NDUFA8, TRAP1, NDUFA7, ATP5PD, NDUFA3, NDUFA1, MRPL24, NDUFAB1]
mitochondrial inner membrane (GO:0005743)	3.48E-20	3.71E-18	[NDUFB9, MRPS15, NDUFA13, NDUFB8, TOMM40, NDUFB7, NDUFA12, MRPS12, MICOS13, PHB, MRPL12, COX5B, PPOX, MTLN, ATP5MC1, TIMM50, MRPL41, MRPL4, ATP5F1A, MRPL2, CYC1, TRAP1, NDUFA8, NDUFA7, MRPS27, ATP5PD, MRPS24, NDUFA3, NDUFA1, MRPS18A, MRPS2, TIMM23, NDUFC2, MRPL24, MRPS5, COX6B1, MRPL52, MRPS9, MRPL51, SLC25A19, NDUFAB1, SLC25A5]
organelle inner membrane (GO:0019866)	2.65E-19	1.88E-17	[NDUFB9, MRPS15, NDUFA13, NDUFB8, TOMM40, NDUFB7, NDUFA12, MRPS12, MICOS13, PHB, MRPL12, COX5B, MTLN, TM7SF2, ATP5MC1, TIMM50, MRPL41, MRPL4, ATP5F1A, MRPL2, CYC1, TRAP1, NDUFA8, NDUFA7, MRPS27, ATP5PD, MRPS24, NDUFA3, NDUFA1, MRPS18A, MRPS2, TIMM23, NDUFC2, MRPL24, MRPS5, COX6B1, MRPL52, MRPS9, MRPL51, SLC25A19, NDUFAB1, SLC25A5]
mitochondrial respiratory chain complex I (GO:0005747)	1.73E-13	7.36E-12	[NDUFB9, NDUFA8, NDUFA13, NDUFB8, NDUFA7, NDUFB7, NDUFA12, NDUFA3, NDUFA1, NDUFC2, NDUFC2-KCTD14, NDUFS8, NDUFS7, NDUFAB1]
respiratory chain complex I (GO:0045271)	1.73E-13	7.36E-12	[NDUFB9, NDUFA8, NDUFA13, NDUFB8, NDUFA7, NDUFB7, NDUFA12, NDUFA3, NDUFA1, NDUFC2, NDUFC2-KCTD14, NDUFS8, NDUFS7, NDUFAB1]
mitochondrial envelope (GO:0005740)	1.49E-07	5.30E-06	[TRAP1, NDUFA8, NDUFA13, NDUFB7, NDUFA1, CHCHD10, TIMM23, ABCB8, PPOX, OXA1L, HAX1, ATP5F1B, NDUFAB1, VDAC2, THOP1]
mitochondrial matrix (GO:0005759)	1.19E-05	3.62E-04	[GSTK1, TRAP1, FH, NDUFB8, NDUFA7, MECR, KARS1, PUS1, MRPS12, ETFB, PDHB, HSD17B10, TXN2, OXA1L, ATP5F1A, MRPL51, ATP5F1B, NDUFS8, GRPEL1, NDUFS7, NDUFAB1, SSBP1]
integral component of mitochondrial inner membrane (GO:0031305)	1.53E-05	4.07E-04	[TMEM177, OXA1L, SAMM50, SLC25A19, TIMM23, CHCHD10, MICOS13, MTLN]
mitochondrial proton-transporting ATP synthase complex (GO:0005753)	3.56E-05	8.42E-04	[ATP5F1A, ATP5PD, ATP5F1B, ATP5MC3, ATP5MC1]
mitochondrial intermembrane space (GO:0005758)	4.01E-05	8.55E-04	[NDUFA8, TRAP1, HAX1, NDUFB7, TIMM23, CHCHD10, PPOX, THOP1]

Supplemental Table S3. Up and downregulated ontology results for the MHS vs Control baseline comparison. Statistically significant (q-value <0.05) GO Biological Process, GO Molecular Function and GO Cellular Component terms are ordered by q-value, which corresponds to the p-value adjusted for multiple comparisons.
