# Supplementary material for: Comparison of Transcriptomic Changes in Survivors of Exertional Heat Illness with Malignant Hyperthermia Susceptible Patients
Source: Int J Mol Sci. 2023 Nov 9;24(22):16124. doi: 10.3390/ijms242216124 (PMC10671540; doi:10.3390/ijms242216124)
Supplement: Supplementary file 1 [file ijms-24-16124-s001.zip › Supplemental Table S4 - baseline Venn Diagram sections.pdf]

Comparison of transcriptomic changes in survivors of exertional heat illness with malignant hyperthermia susceptible patients

Supplemental Table S4. Venn diagram segments

UP in EHI and MHS (51)	DOWN in EHI and MHS (33)	Only UP in MHS (524)								
AGAP5	ABI3	ABCA1	CACNA1D	DMXL2	GOLGA8Q	LINC00680-GUSBP4	NUP50	REV3L	STAU2	WDFY1
ASB7	ADA	ABCB1	CASC4	DNAJC16	GOLGA8R	LINC01303	OSBPL11	RFFL	STIM2	WDR19
ATXN7	ALG8	ABCC1	CCDC170	DOCK11	GOLGA8T	LMTK2	PACC1	RFWD3	STK4	WDR44
B3GNT2	ANAPC5	ABHD17B	CCDC191	DOCK4	GOLIM4	LNX2	PALLD	RIC1	STX11	WDR47
BAZ1A	APEX1	ACBD5	CCDC93	DOCK9	GOSR1	LOC100287042	PANX1	RLF	SUCO	WDR7
CASP8AP2	ATF5	ACOT11	CCP110	DSC2	GPATCH8	LOC728752	PAXIP1	RNASEL	SUDS3	WHAMM
CFAP58-DT	CCT3	ACSL4	CD274	DSTYK	GPBP1L1	LPGAT1	PCF11	RNF111	SUSD6	WHAMMP1
CFH	FH	ACTR8	CDC14A	DYRK1A	GPD2	LRCH1	PCM1	RNF144A	SYNE1	XYLT1
CMTM1	GAR1	ADAM10	CDC5L	E2F3	GSE1	LRIF1	PDCD7	RNF145	SYNE2	ZBED4
DAPP1	GPATCH4	ADCY7	CDK13	EAF1	HEATR5A	LRRC1	PDE3B	RNF168	TAB2	ZBTB11
DDX59	IMP4	ADD3	CDK17	EBLN2	HEATR5B	LRRFIP1	PDPK1	RNF6	TAF1	ZBTB38
ECT2	KARS1	ADNP	CDK5R1	EDEM1	HECTD1	LY75	PDS5B	ROCK1	TAF2	ZC3H11A
ERLIN2	LPXN	ADNP2	CDYL	EDEM3	HECTD4	MACF1	PER3	RREB1	TAF5L	ZC3H13
F2RL1	MC1R	AFF1	CEACAM1	EFR3A	HEG1	MAF	PFKFB2	RTL6	TASOR2	ZFP36L2
FCHO2	MIR1244-1	AGAP1	CEMIP2	ELF1	HERC2P3	MAML1	PHF2	SBF2	TBC1D14	ZFP91-CNTF
GTF2E1	MIR1244-2	AGTPBP1	CEP120	ELF2	HERC2P9	MAP3K1	PHLPP2	SBNO1	TBC1D2B	ZFR
IFIT2	MIR1244-3	AKAP9	CEP170	ELK3	HERPUD2	MAP3K7	PHTF1	SCAF11	TDRD7	ZKSCAN1
IL18R1	MIR1244-4	AMMECR1L	CEP170P1	EML5	HIF1A	MAST4	PIK3AP1	SCAF8	TENT4B	ZKSCAN4
ITPRID2	MRPS9	ANGPT1	CEP295	EMSY	HIPK1	MBNL2	PIK3CG	SDE2	TET2	ZMYND11
JADE1	NCR3	ANKIB1	CEP350	EPC2	HIPK2	MBTPS1	PIKFYVE	SECISBP2L	TET3	ZMYND8
KAT2B	NOL10	ANKRD11	CEP85	EPG5	HK2	MCM9	PIP5K1A	SEMA3C	TFDP2	ZNF106
KCNJ2	RBM4	ANKRD13C	CGAS	EPHA4	HMGCR	MED13L	PJA2	SENP6	TGFA	ZNF189
KLHL12	RHOC	ANKRD27	CHD1	EPS15	HMGXB4	MED23	PLCB1	SETD2	TGFBR1	ZNF217
KLHL2	SAMM50	ANKRD33B	CHD2	ERBIN	HOXB3	MEGF9	PLCXD2	SETD5	TGFBR2	ZNF236
KTN1	SGF29	AP4E1	CHD6	ERMP1	HP1BP3	MERTK	PLEKHA2	SETX	TIRAP	ZNF280C
LDLRAD4	SNORA100	APC	CHD9	ETV3	HTATSF1	METTL15	PLEKHM3	SF3B1	TMED7-TICAM2	ZNF281
LINC00243	SNORD35B	APPL2	CIPC	F2R	INPP4A	MEX3C	PLXNC1	SFMBT1	TMEM181	ZNF292
LINC00667	TCAP	AREL1	CLASP2	FAM120A	IQGAP2	MFAP3	PNN	SH3BP5	TMOD2	ZNF304
LOC101930085	TEX10	ARHGAP19-SLIT1	CLINT1	FAM13A	ITGB1	MFSD6	PODXL	SHPRH	TNFAIP3	ZNF395
MIR5047	TIMM50	ARHGEF3	CLIP1	FAM160B1	ITPR1	MGA	POLD3	SIAH1	TNRC6A	ZNF45
MIR6125	UTP14A	ARID1B	CMTM4	FAM161B	ITSN2	MICB	PPARA	SLAIN2	TNRC6B	ZNF468
NPTN-IT1	VDAC2	ARID4A	CNNM4	FAM168A	IVNS1ABP	MIDEAS	PPFIA1	SLC19A2	TNRC6C	ZNF507
PDE4B	VPS45	ARID4B	CNOT6	FAM172A	JAG1	MIR3652	PPM1B	SLC23A2	TOGARAM1	ZNF585A
PDP1		ARSB	CNST	FBXO33	JMJD1C	MIR6501	PPP1R12A	SLC24A1	TOPBP1	ZNF611
PELI1		ASH1L	CNTRL	FBXO34	KAT6B	MIR6506	PPP6R3	SLC8A1	TOR1AIP1	ZNF614
PHF20L1		ASXL2	COG3	FBXW2	KBTBD6	MKRN2	PRDM1	SLCO4C1	TP53BP2	ZNF619
PLSCR1		ATAD2B	CPD	FBXW7	KBTBD7	MTM1	PRDM10	SLFN13	TRAPPC10	ZNF638
PPP4R1L		ATE1	CPEB4	FLI1	KCTD20	MTMR1	PRKAR1A	SLFN5	TRIM56	ZNF644
PRKAG2		ATG16L1	CR1	FNDC3A	KDM3A	MTMR4	PRKAR2A	SLK	TSPYL1	ZNF669
PWWP2A		ATL3	CRACR2A	FNDC3B	KDM6A	MTR	PRMT9	SLMAP	TTL	ZNF674
RP2		ATP11A	CRAMP1	FNIP2	KIAA0355	MYO5A	PRPF38B	SLTM	TTN-AS1	ZNF808
SERINC5		ATP2B1	CREB3L2	FOXO3B	KIAA0753	MYOM1	PSME4	SLX4IP	TTPAL	ZSCAN29
SLC40A1		ATP7A	CRLF3	FRMD4B	KIAA1109	N4BP2	PTCH1	SMAD7	TUT7	ZSCAN30
SP3		ATP8A1	CRY1	FRY	KIDINS220	NCBP3	PTGER4	SMARCA5	UBQLN1	ZSWIM6
TMEM165		ATRX	CSPP1	FUBP3	KIF16B	NCEH1	PTGS2	SMC3	UBR3	
TMEM68		ATXN1L	CTNNB1	FUT11	KIF21A	NCOA2	PTPN11	SMCHD1	UBR5	
TMF1		AUTS2	CYLD	FYCO1	KIF5B	NCOA7	PTPRC	SMURF1	UGDH	
TNFAIP6		B3GNT5	CYSLTR2	FZD6	KIFAP3	NEK9	PTPRE	SMURF2	UPF2	
TOB1		BACH1	DAAM1	GAB1	KIT	NFYA	PUM1	SNRK	USP25	
TRIM22		BAZ2B	DBT	GALC	KLHL20	NIN	QTRT2	SNX18	USP30	
ZNF624		BDP1	DCP1A	GALNT3	KLHL36	NINL	R3HDM2	SOCS7	USP38	
		BIRC6	DCTN4	GBP1	KMT2A	NIPA1	RAB3GAP2	SOS2	USP42	
		BMP8B	DDI2	GCC2	KMT2C	NKRF	RAD21	SPART	USP47	
		BMS1P4-AGAP5	DDX3X	GLCCI1	KMT2E	NKTR	RAD51-AS1	SPATA13	UTP14C	
		BOD1L1	DENND4C	GNPTAB	L3MBTL4	NR2C2	RALGAPB	SPIN4	VAV3	
		BPTF	DHFR2	GOLGA4	LATS2	NR3C1	RB1CC1	SPTY2D1	VEZF1	
		BRCA1	DIP2A	GOLGA8B	LAX1	NR3C2	RBM27	SRGAP2B	VPS13B	
		BRD1	DIP2B	GOLGA8J	LBR	NSD2	RBMXL1	SRGAP2D	VPS13C	
		C20orf194	DIP2C	GOLGA8N	LIN54	NUP153	RECK	SSH1	VPS4B	
		C9orf129	DLG1	GOLGA8O	LINC00680	NUP160	RESF1	STAM	WAPL	

Only DOWN in MHS (411)							Only up in EHI (102)		Only down in EHI (105)	
ABCB8	CUTA	HAUS7	MRPL41	PAFAH1B3	RNA45SN2	THOC6	AFF1-AS1	NOTCH2NLA	ACSS1	NKG7
ABHD14A	CYB561D2	HAX1	MRPL51	PARK7	RNA45SN3	THOP1	ANKRD49	OMG	AHSA1	NOP2
ACBD4	CYC1	HBG1	MRPL52	PAXX	RNA45SN4	TIMM23	ANXA3	OSM	ARL17B	OSBPL5
AKR1A1	DAD1	HCFC1R1	MRPS12	PCED1A	RNA45SN5	TM7SF2	ARGLU1	PDCD10	ARVCF	PDZD4
AKR1B1	DBH-AS1	HCST	MRPS15	PCF11-AS1	RNA5-8SN1	TMED3	ARRDC3	PKHD1L1	ATIC	PLEKHF1
ALG3	DBNDD2	HIGD2A	MRPS18A	PDHB	RNA5-8SN2	TMEM138	BCL2A1	POLR2M	C11orf24	POU2F2
ANAPC11	DCANP1	HLA-DMA	MRPS2	PEMT	RNA5-8SN3	TMEM141	BTG2	PPM1A	C2orf42	PPM1G
ANKS3	DNPH1	HLA-DPA1	MRPS24	PEX11B	RNA5-8SN4	TMEM147	C11orf54	PSMC6	CACNA2D2	PRF1
AP1S1	DPP7	HLA-DRB1	MRPS27	PFDN6	RNA5-8SN5	TMEM177	CACNB4	PSMD10	CACNB1	PRPF19
ARFIP2	DRG1	HLA-J	MRPS5	PHB	RNASEH2C	TMEM186	CAPZA2	PTPRN2	CCT7	PSMB2
ARL6IP4	DUS1L	HSD11B1L	MT1E	PHF11	RNF181	TMEM222	CARD17	RAB28	CCT8	PTDSS1
ARMCX6	ECHDC2	HSD17B10	MT1X	PHPT1	RNF5	TMEM223	CASP3	RANBP6	CD247	RASSF1
ARRDC1-AS1	EEF1AKMT4	HYLS1	MTLN	PIH1D1	RPL14	TMEM258	CAVIN2	RGS18	CD81	RBBP7
ATP5F1A	EEF1AKMT4-ECE2	HYPK	MVK	POLD2	RPL36	TMEM60	CD300LD	RHNO1	CDK18	RGS9
ATP5F1B	EGFL8	ICAM2	MYBPH	POLR1C	RPLP2	TMSB10	CD69	RNF103-CHMP3	CHST10	RUVBL1
ATP5MC1	EIF3G	ICAM4	MYL5	POLR2G	RPP21	TMX2	CENPQ	RNF11	CLDND2	S1PR5
ATP5MC3	EIF4A1	ILF2	MYL6B	POMGNT1	RPP25	TOMM40	CHMP3	RNF141	CLIC3	SAMD1
ATP5PD	EIF5A	ILKAP	NAA10	POMP	RPP38	TRAP1	CHMP5	RPS18P9	CNOT10	SARS1
ATRAID	ELOA-AS1	ILVBL	NAALADL1	POMT1	RPS6KB2	TRAPPC3	CHSY1	SAMSN1	CNOT11	SEPTIN6
AUP1	ELP4	IMPDH2	NAT14	POP5	RRAS	TRIM39-RPP21	CKMT2-AS1	SLC35G1	COL6A2	SKAP1
B3GAT3	ELP5	KCTD17	NAT9	PPIB	RRP9	TRMT1	CTNNAL1	SLC37A3	CPSF3	SLC43A1
B4GALT3	EMC9	KIFC2	NDUFA1	PPOX	RTCB	TRMT2A	CXCL5	SLU7	CSTF2	SLC5A6
B4GALT7	EMP3	KRT10	NDUFA12	PPP1R3E	RUSC1-AS1	TRMT61A	DNAJC25-GNG10	SRGN	CTSW	SLC9A3-AS1
BANF1	ERCC1	KRTCAP2	NDUFA13	PPT2-EGFL8	S100A10	TRMU	DNAJC3-DT	TAX1BP1	CXXC5	SMAGP
BCL7C	ERP29	LAGE3	NDUFA3	PRDX2	SAPCD1	TSPAN17	DSE	TCAIM	DDX47	SOX12
BUD23	ESD	LGALS1	NDUFA7	PRMT7	SAT2	TXN2	EDA	TFDP1	DHCR24	SPINDOC
C14orf119	ETFB	LGALS4	NDUFA8	PROC	SDF2L1	U2AF1L4	EID2B	TMEM185B	DPAGT1	STIP1
C17orf49	EXOSC1	LIN7B	NDUFAB1	PRR22	SDR39U1	UBC	EIF4E3	TMX1	DTX3	STT3A
C19orf48	EXOSC5	LINC00861	NDUFB7	PSMA5	SEC61B	UBE2CP5	EXPH5	TNFSF13B	ERBB2	TAF15
C19orf53	FADS2	LINC01503	NDUFB8	PSMB5	SEC61G	URGCP-MRPS24	F8	TTC33	EXOSC10	TBX21
C1orf122	FAM160B2	LMF2	NDUFB9	PSMB6	SELENOM	URM1	FGFR1OP2	TWF1	FAF1	TCP1
C1orf162	FAM207A	LOC114841035	NDUFC2	PSMB7	SENP3-EIF4A1	UROD	FOPNL	ZFP37	FEZ1	TESK1
C2orf92	FAM3A	LOC200772	NDUFC2-KCTD14	PSMC3	SERINC2	USE1	GET1-SH3BGR	ZNF140	FIG4	TKTL1
CCDC107	FAM50A	LRRC24	NDUFS7	PSMC5	SHLD1	VPS25	GIN1	ZNF230	GARS1	TMEM109
CCDC154	FARSB	LSM12	NDUFS8	PSMD8	SLC16A11	WDR74	GNG10	ZNF267	GNLY	TMEM189-UBE2V1
CCDC78	FASTK	LY6G5B	NENF	PSMG2	SLC25A19	WDR83OS	GOLGA7	ZNF441	GORASP2	TPST2
CCDC84	FBXO44	LY86	NEURL1	PTPRCAP	SLC25A5	YIF1A	GPBP1	ZNF443	GPR68	TTC38
CCZ1P-OR7E38P	FCER1G	LYNX1	NHP2	PUS1	SLC2A6	YJU2	GPR27	ZNF484	GZMB	UBE2V1
CDH23	FGFRL1	LYPD2	NIT2	RAB33A	SNHG1	ZBED5-AS1	GRAMD2B	ZNF627	HARS1	UTP4

CDK10	FHIT	LZTR1	NME1	RAB3IL1	SNHG16	ZDHHC16	GUSBP9	ZNF658B	HNRNPA3	VDAC1
CDK4	FKBP2	LZTS3	NMRAL1	RANBP1	SNHG20	ZNF263	H2AC6	ZNF736	HNRNPD	WDR46
CENPT	FLAD1	MACROD1	NOP56	RANGRF	SNORA10	ZNF511	H4C14	ZNF844	IGFBP4	YARS1
CENPX	GABBR1	MALSU1	NOSIP	REEP6	SNORD36A	ZNF513	H4C15		IL21R	YTHDF2
CETN2	GAMT	MAMDC4	NPIPA1	RELL2	SNRPC	ZNF517	HMGB2		IL27RA	ZNF296
CHCHD10	GATD1	MAN1B1	NPIPA5	RHPN1	SPATC1L	ZNF524	IFI44		IL2RB	ZNNT1
CHMP4A	GEMIN7	MCRIP2	NPM3	RING1	SPCS1	ZNF561-AS1	LINC00654		KDM1A	
CIAO2B	GFER	MECR	NR2C2AP	RN7SL1	SPIN2B	ZNF580	LMNB1		KPNA2	
CIAO3	GIMAP1-GIMAP5	METTL1	NR2F6	RN7SL2	SSBP1	ZNF582-AS1	LOC101928429		LAIR1	
CINP	GIMAP5	METTL26	NSUN5P2	RN7SL3	SSNA1	ZNF593	LRP11		LINC01278	
CISD3	GLI4	MGST3	NT5C	RNA18SN1	STARD10	ZNRD2	LSM8		LLGL2	
CLEC10A	GMPPB	MICOS13	NT5DC2	RNA18SN2	STK32C	ZSCAN16-AS1	MCEE		LMNB2	
CNIH2	GRPEL1	MIR12136	NTHL1	RNA18SN3	SUMO3		MFSD14B		LOC100129083	
CNPY4	GSDMD	MIR142	NUTF2	RNA18SN4	SWI5		MLH3		LOC100505501	
COPS6	GSTK1	MIR611	OCM	RNA18SN5	SYT17		MMD		MATK	
COPZ1	GTF2A2	MIR7705	ORMDL2	RNA28SN1	TAGLN		MTRR		MEAK7	
COX5B	GTF2H4	MPDU1	OSGEP	RNA28SN2	TARBP2		MYNN		MLC1	
COX6B1	GUSBP11	MRPL12	OST4	RNA28SN3	TBCB		NAT1		MMP23B	
CPSF4	H2AC8	MRPL2	OVCA2	RNA28SN4	TCEA2		NEBL		MTCH2	
CRIP3	HACL1	MRPL24	OXA1L	RNA28SN5	TCEA3		NEK7		N4BP2L2-IT2	
CRYGS	HAGHL	MRPL4	P2RX4	RNA45SN1	THAP7		NFYB		NAB2	

Supplemental Table S4. All genes allocated to each venn diagram segment is displayed in alphabetical order.
