# Supplementary material for: Comparison of Transcriptomic Changes in Survivors of Exertional Heat Illness with Malignant Hyperthermia Susceptible Patients
Source: Int J Mol Sci. 2023 Nov 9;24(22):16124. doi: 10.3390/ijms242216124 (PMC10671540; doi:10.3390/ijms242216124)
Supplement: Supplementary file 1 [file ijms-24-16124-s001.zip › Supplemental Table S5 - Timepoint hallmarks.pdf]

Comparison of transcriptomic changes in survivors of exertional heat illness with malignant hyperthermia susceptible patients

Supplemental Table S5. Timepoint Hallmarks

Upregulated MSigDB Hallmarks at 2 hours post HTT vs Pre

term	p-value	q-value	overlap_genes
TNF-alpha Signaling via NF-kB	2.97E-17	1.49E-15	[BTG2, BTG1, TNFAIP6, PLEK, TNFAIP2, RNF19B, CXCL1, SLC2A3, LITAF, TNF, SAT1, ETS2, IFIT2, ZFP36, PLAU, NAMPT, SLC16A6, PDE4B, DENND5A, CCNL1, JUNB, IER2, MAP2K3, KDM6B, DUSP1, IFNGR2, IL18, PLAUR, FOS, SERPINB8, RHOB, IL1B, NINJ1, TRIB1, PLPP3, SGK1, BIRC2, TLR2, B4GALT5, PPP1R15A, NFAT5, CEBPB, PFKFB3, BCL2A1, CEBPD, PTGS2, TANK, SOCS3, SNN, DRAM1, NFIL3, MCL1, ABCA1, EGR1, EGR3, GADD45B, LAMB3, TNFRSF9, G0S2, CFLAR, ATP2B1, SOD2, PNRC1, KLF2, FOSL2, VEGFA, PTPRE, KLF6, MARCKS, BCL6, BCL3, F2RL1, MXD1, NFE2L2]
Inflammatory Response	3.66E-08	9.15E-07	[BTG2, CSF3R, TNFAIP6, AQP9, ADM, BEST1, IL18RAP, NAMPT, CLEC5A, TNFSF10, PDE4B, PROK2, FFAR2, KIF1B, IL10, IL4R, IL1R1, IFNGR2, IL18, RHOG, PLAUR, TNFRSF1B, TLR1, IL1B, LCP2, RAF1, TLR2, IFNAR1, PTAFR, C5AR1, FPR1, LPAR1, GNAI3, PSEN1, SRI, NOD2, MEFV, HIF1A, PIK3R5, CD55, KCNJ2, LYN, ABCA1, SLC31A2, SLC31A1, TNFRSF9, OSM, EIF2AK2, NMI, ATP2B1, PTPRE, KLF6, SELL, ABI1, MXD1, IL18R1]
Complement	2.35E-07	3.91E-06	[DGKG, CDA, DOCK4, SERPINA1, ITGAM, CPQ, PLEK, CXCL1, CTSS, PIK3CG, CASP9, AKAP10, LAMP2, CASP4, TIMP2, CASP1, CD36, USP8, CR1, PRKCD, RHOG, PLAUR, PLA2G4A, DUSP6, F5, PLSCR1, GPD2, IRF2, ADAM9, LCP2, CD46, RAF1, S100A9, USP15, CEBPB, GNAI3, PRCP, PSEN1, PIK3R5, PPP4C, GNG2, S100A12, CD59, CD55, LYN, GCA, BRPF3, RABIF, GNB2, GNB4, GRB2, CDK5R1, MSRB1, HSPA1A]
IL-6/JAK/STAT3 Signaling	4.60E-07	5.75E-06	[CSF3R, CXCL1, CSF2RB, CBL, TNF, CSF2RA, PIK3R5, SOCS3, CD36, CCR1, IL4R, TNFRSF12A, IL1R1, IFNGR1, IL10RB, IFNGR2, IL1R2, STAT3, TYK2, TNFRSF1B, TNFRSF1A, IL17RA, IL1B, FAS, GRB2, STAM2, MYD88, IL18R1, TLR2, IFNAR1]
Interferon Gamma Response	3.16E-06	3.16E-05	[IFITM2, BTG1, EIF4E3, TNFAIP6, TNFAIP2, ADAR, IFIT3, IFIT2, CASP8, NAMPT, CASP4, TNFSF10, CASP1, PDE4B, TRIM25, TRIM21, GBP6, ZBP1, IFNAR2, IL4R, SP110, HLA-B, TRAFD1, PLA2G4A, PLSCR1, IRF2, PELI1, LCP2, RBCK1, MVP, FGL2, FPR1, CSF2RB, SRI, PTGS2, HIF1A, HELZ2, SOCS3, ST8SIA4, FCGR1A, MX2, STAT3, LYSMD2, EIF2AK2, TDRD7, NMI, TOR1B, SOD2, PSMB8, FAS, MYD88]
PI3K/AKT/mTOR Signaling	4.21E-06	3.51E-05	[GSK3B, CDKN1B, RALB, CAB39, YWHAB, UBE2D3, CLTC, PTEN, SLA, DAPP1, CXCR4, TBK1, MKNK1, RPS6KA1, MKNK2, MAPK1, RAC1, MAP2K6, MAP2K3, ACTR3, VAV3, ACTR2, DUSP3, PRKCB, IRAK4, TNFRSF1A, RIT1, ARPC3, DDIT3, GRB2, RAF1, MYD88]
Apoptosis	1.24E-05	8.88E-05	[BTG2, RNASEL, CDKN1B, ROCK1, HMGB2, NEDD9, BCL10, PSEN1, BRCA1, TNF, SAT1, CASP9, PPP3R1, PAK1, CASP8, CASP4, TNFSF10, TIMP2, CASP1, PMAIP1, BID, MCL1, BNIP3L, CREBBP, EGR3, GADD45B, TNFRSF12A, GPX3, IFNGR1, IL18, CFLAR, SOD2, IGF2R, RHOB, DNAJC3, DDIT3, IL1B, RARA, FAS, CTNNB1, ETF1, PLCB2]
Protein Secretion	1.08E-04	6.78E-04	[STX12, CD63, TSG101, SGMS1, STX16, COPB1, VPS4B, CLTC, SNAP23, AP1G1, LAMP2, MAPK1, KIF1B, ARFIP1, ATP7A, YKT6, RAB2A, ARFGEF1, ABCA1, STX7, ADAM10, IGF2R, RER1, YIPF6, SEC22B, RAB5A, VAMP3]
Reactive Oxygen Species Pathway	1.39E-03	7.71E-03	[CDKN2D, EGLN2, SRXN1, GPX3, LSP1, OXSR1, TXN, IPCEF1, SOD2, MSRA, HHEX, SBNO2, MBP, JUNB, LAMTOR5]

Interferon Alpha Response	1.87E-03	7.92E-03	[IFITM2, IL4R, SAMD9, SP110, HLA-C, TRAFD1, EIF2AK2, TDRD7, ADAR, NMI, TMEM140, PARP9, IFIT3, PSMB8, IFIT2, HELZ2, PLSCR1, CASP8, SELL, IRF2, CASP1, TRIM25, GBP2, TRIM21]

Downregulated MSigDB Hallmarks at 2 hours post HTT vs Pre

term	p-value	q-value	overlap_genes
Myc Targets V2	2.46E-17	1.23E-15	[NOP2, PPAN, NOLC1, NOC4L, TMEM97, PHB, IPO4, RRP9, SRM, TBRG4, UNG, EXOSC5, TCOF1, PES1, UTP20, NOP56, DUSP2, NOP16, PUS1, WDR74, IMP4, PA2G4, PPRC1, SUPV3L1, AIMP2, MYBBP1A, LAS1L, CDK4, GRWD1, MRTO4, MCM4, FARSA, DCTPP1, SLC29A2]
Myc Targets V1	1.93E-10	4.81E-09	[MCM7, HNRNPR, RRP9, FBL, YWHAQ, RUVBL2, PSMD3, MRPL9, KPNA2, CCT3, STARD7, PABPC4, CAD, MRPS18B, CTPS1, ILF2, NME1, TCP1, NHP2, MCM4, VDAC1, MCM6, SNRPA, MCM2, SF3B3, KARS1, CSTF2, CUL1, NOLC1, PHB, GSPT1, SRM, EXOSC7, PSMB2, G3BP1, POLD2, CYC1, IARS1, CCT7, HNRNPA1, CCT4, NOP56, RANBP1, RRM1, NOP16, GOT2, PA2G4, AIMP2, RSL1D1, EPRS1, CDK4, APEX1, SERBP1, POLE3, RNPS1, PRPF31, EIF3B]
mTORC1 Signaling	3.62E-08	6.04E-07	[GPI, TES, CCNF, SLC2A1, ATP2A2, TMEM97, RRP9, ME1, SLC37A4, FADS1, ELOVL6, TUBG1, SLC7A5, DDX39A, DDIT4, HMBS, MCM4, MCM2, NUP205, TOMM40, SHMT2, GMPS, PIK3R3, SLC1A5, ACACA, UNG, HSP90B1, ATP5MC1, STIP1, EBP, SERPINH1, ABCF2, LDLR, HSPA9, CYB5B, XBP1, SDF2L1, GOT1, CYP51A1, RPA1, M6PR, IMMT, DHCR24, MLLT11, CCT6A, QDPR, PFKL, EPRS1, SCD, CALR, DHCR7, LGMN]
Unfolded Protein Response	6.13E-08	7.66E-07	[H2AX, GOSR2, PARN, NOLC1, RRP9, HSP90B1, EXOSC5, TATDN2, EXOSC10, IARS1, SEC31A, TSPYL2, HSPA9, NOP56, XBP1, NOP14, IMP3, WFS1, DDX10, EEF2, PDIA6, LSM4, PDIA5, SLC7A5, DKC1, DDIT4, NHP2, GEMIN4, SRPRB, PREB, HYOU1, CALR, ALDH18A1, TARS1, ATF3]
E2F Targets	1.51E-06	1.51E-05	[H2AX, PRPS1, NUP205, DNMT1, RNASEH2A, MCM7, SHMT1, SUV39H1, NOLC1, BUB1B, MKI67, GSPT1, UNG, TBRG4, POLD1, STMN1, POLD2, RBBP7, KPNA2, LYAR, TCF19, NOP56, RANBP1, CBX5, LIG1, TUBB, GINS3, RPA1, CTPS1, PA2G4, KIF22, SSRP1, TUBG1, PAICS, NME1, CDC25B, ILF3, DDX39A, NASP, CCNE1, CDK4, MCM3, MCM4, MCM6, TP53, DCTPP1, SNRPB, MCM2]
IL-2/STAT5 Signaling	6.49E-04	5.41E-03	[CDKN1C, NRP1, RNH1, ABCB1, AHNAK, CD81, PTGER2, NOP2, SLC1A5, PDCD2L, IL1RL1, CCND2, SOCS1, NCS1, TNFSF11, SNX9, DCPS, EOMES, XBP1, PRKCH, CISH, IL10RA, PUS1, PTCH1, TNFRSF18, HUWE1, FLT3LG, RHOH, DHRS3, HOPX, SERPINB6, UCK2, MYO1C, CCNE1, SYT11, IL2RB, BCL2, IRF8, TNFRSF21, SLC29A2]
Cholesterol Homeostasis	8.90E-04	6.36E-03	[PCYT2, MVK, CHKA, CYP51A1, TMEM97, LSS, SREBF2, ACAT2, EBP, SCD, FASN, PMVK, MVD, DHCR7, GUSB, ATF5, LDLR, ATF3, LGMN]
Oxidative Phosphorylation	1.37E-03	8.55E-03	[GPI, FH, NDUFB8, ECHS1, MRPS12, TIMM13, ATP5MC3, HSD17B10, ATP5MC1, TIMM50, ATP5F1A, ATP5F1B, GRPEL1, AIFM1, PMPCA, CYC1, NDUFV1, COX10, IDH3A, HSPA9, NDUFA9, NDUFA8, PDHA1, MDH2, IDH2, GOT2, IMMT, SDHA, CS, SUPV3L1, AFG3L2, ALDH6A1, NDUFS8, VDAC1, ACO2, SLC25A12, SLC25A4, FXN, SLC25A6]
DNA Repair	1.57E-03	8.72E-03	[FEN1, RNMT, USP11, NT5C, SEC61A1, ZNF707, TAF1C, POLD1, POLR2D, DGCR8, NELFCD, POLH, LIG1, PDE6G, SSRP1, TARBP2, DDB2, NME1, APRT, DDB1, RAD51, ERCC3, NFX1, POLR3C, ERCC1, POLR1C, ERCC2, ERCC5, TP53, ITPA, ADA]
Fatty Acid Metabolism	6.79E-03	3.40E-02	[GCDH, FH, ECHS1, ECI2, RAP1GDS1, HSD17B10, ACAT2, HSPH1, NTHL1, ME1, D2HGDH, HADH, ACSS1, PDHA1, MDH2, UROS, ACSL5, DHCR24, MIF, SDHA, ALDH3A2, GSTZ1, FASN, APEX1, ACOT2, ACO2, REEP6, CRAT, MGLL, METAP1]

Upregulated MSigDB Hallmarks at 24 hours post HTT vs 2 hours

term	p-value	q-value	overlap_genes
G2-M Checkpoint	5.65E-09	2.83E-07	[TOP2A, CCNT1, KIF11, MKI67, TENT4A, SMC4, SMC2, KIF15, SYNCRIP, SNRPD1, NEK2, FBXO5, TNPO2, TMPO, SS18, SLC38A1, ATRX, HUS1, PRPF4B, LIG3, KNL1, SMC1A, ILF3, SFPQ, RBL1, DBF4, INCENP, DKC1, NCL, MTF2, CKS2, SRSF2, MCM3, MCM6, KIF20B, MCM2, CUL5, SRSF1, CUL1, NOLC1, TTK, SLC7A1, PURA, WRN, ORC6, TRA2B, NSD2, G3BP1, ABL1, RAD54L, SRSF10, BARD1, SLC12A2, CASP8AP2, HSPA8, POLQ, STIL, SMAD3, FANCC, MEIS2, CUL4A, SQLE, CENPE, UCK2, CENPF, NASP, PRC1, MNAT1, EZH2]
E2F Targets	4.59E-07	1.15E-05	[TOP2A, NUP107, MRE11, TFRC, CSE1L, CCP110, BUB1B, PSIP1, SMC6, SMC3, MKI67, SMC4, IPO7, SYNCRIP, TMPO, HELLS, RFC1, HUS1, CTPS1, SMC1A, DCK, CIT, MMS22L, ILF3, MSH2, MTHFD2, DEPDC1, CKS2, SRSF2, MCM3, MCM4, LUC7L3, MCM6, ASF1A, MCM2, PRPS1, NUP205, DNMT1, SRSF1, NOLC1, ORC6, TRA2B, PMS2, RBBP7, LYAR, BARD1, CBX5, GINS3, ATAD2, RPA1, NAP1L1, PAICS, EIF2S1, CENPE, NUDT21, RAD50, WEE1, DIAPH3, NASP, UBE2T, BRMS1L, ANP32E, RAD1, EZH2]
Unfolded Protein Response	2.72E-05	4.54E-04	[EIF4A2, FKBP14, GOSR2, PARN, NOLC1, SDAD1, HSP90B1, BAG3, EXOSC9, DNAJB9, MTREX, IARS1, EXOSC2, PAIP1, HSPA9, TTC37, NOP14, XBP1, NPM1, NFYB, SLC30A5, EDEM1, EIF2AK3, DDX10, PDIA6, EIF2S1, PDIA5, XPOT, MTHFD2, DKC1, PSAT1, DDIT4, SRPRB, HYOU1, ALDH18A1, DCP1A, TARS1, EXOC2]
Myc Targets V1	5.54E-04	6.92E-03	[RPL34, HNRNPR, PWP1, SYNCRIP, YWHAQ, SNRPD1, DHX15, ACP1, TOMM70, DDX18, CCT2, NCBP1, STARD7, EIF1AX, NCBP2, CTPS1, GNL3, CLNS1A, CANX, SRSF2, MCM4, SNRPA1, MCM6, SRSF7, MCM2, PRPS2, SET, SF3B3, GLO1, SRSF1, CUL1, NOLC1, DDX21, HSPD1, TRA2B, G3BP1, IARS1, HNRNPA1, CCT4, NPM1, RRM1, HNRNPA3, SSB, NAP1L1, EIF2S1, RSL1D1, EPRS1, XPOT, SERBP1, POLE3, EIF3J, UBA2, TARDBP, ABCE1]
Mitotic Spindle	8.74E-04	8.74E-03	[TOP2A, CEP57, TRIO, ITSN1, KIF11, WASL, SMC3, ARHGAP5, SMC4, KIF15, PCM1, OPHN1, CNTRL, TLK1, KNTC1, NEK2, FBXO5, KIFAP3, ARHGEF12, RFC1, DST, SMC1A, CKAP5, CD2AP, LATS1, TIAM1, ALS2, INCENP, RASA1, ARHGEF3, KIF20B, SOS1, RAPGEF6, RABGAP1, TTK, ABL1, CEP192, FLNB, PCNT, SPTBN1, DYNC1H1, ARHGAP29, SORBS2, LRPPRC, NET1, CCDC88A, CENPE, MYO1E, CENPF, DLG1, PRC1, ALMS1, TUBGCP5]
mTORC1 Signaling	2.94E-03	2.45E-02	[TES, TFRC, PITPNB, ATP2A2, SLC7A11, ME1, FADS1, PDK1, STARD4, HMGCS1, ELOVL6, UCHL5, SYTL2, PPA1, MTHFD2, DDIT4, CANX, MCM4, MCM2, NUP205, NUFIP1, INSIG1, USO1, GMPS, PIK3R3, ADD3, ACACA, HSP90B1, HSPD1, RDH11, SC5D, LDLR, HSPA9, CYB5B, XBP1, GOT1, PNO1, EDEM1, CYP51A1, RPA1, M6PR, DHCR24, CCT6A, MLLT11, SQLE, GCLC, UFM1, EPRS1, SCD, PSAT1, TCEA1]
UV Response Dn	5.13E-03	3.66E-02	[NRP1, INSIG1, PIK3R3, PTPRM, TENT4A, ATP2C1, ADD3, KALRN, SLC7A1, SYNE1, AKT3, ERBB2, TOGARAM1, FYN, LDLR, PDGFRB, DMAC2L, ABCC1, SMAD3, MIOS, SFMBT1, PRKCE, BCKDHB, ATRX, PRKCA, NR1D2, SMAD7, TGFBR3, INPP4B, ATRN, DLG1, ARHGEF9, COL5A2, KIT, PMP22, AGGF1, CDK13, BMPR1A]
Androgen Response	1.21E-02	7.58E-02	[SLC26A2, TNFAIP8, INSIG1, STK39, UAP1, ELK4, AKAP12, LMAN1, ANKH, ADAMTS1, PGM3, DNAJB9, ITGAV, FADS1, TPD52, ABCC4, RAB4A, HMGCS1, ZBTB10, ARID5B, DHCR24, INPP4B, MAF, CDK6, SCD, SELENOP, GPD1L]
IL-2/STAT5 Signaling	2.70E-02	1.50E-01	[NRP1, PHTF2, ABCB1, BMPR2, AHNAK, PTGER2, RORA, AHR, IKZF2, IKZF4, SOCS2, IL1RL1, ALCAM, CCND2, SOCS1, LRIG1, LRRC8C, TNFSF11, SNX9, SLC39A8, ITGAV, EOMES, PRNP, XBP1, PRKCH, IL10RA, PTCH1, HUWE1, RHOH, HOPX, HIPK2, TTC39B, TIAM1, CXCL10, MYO1E, UCK2, IRF4, SYT11, IL2RA, IL2RB, BCL2, IRF8, ITGA6, UMPS, TNFRSF21, CDC42SE2]
Myc Targets V2	3.85E-02	1.92E-01	[DDX18, DUSP2, NPM1, NIP7, PRMT3, NOLC1, TFB2M, WDR43, GNL3, HSPD1, SUPV3L1, RCL1, MYBBP1A, MCM4, MPHOSPH10, UTP20]

Downregulated MSigDB Hallmarks at 24 hours post HTT vs 2 hours

term	p-value	q-value	overlap_genes
TNF-alpha Signaling via NF-kB	1.67E-09	8.11E-08	[BTG2, CDKN1A, B4GALT1, TNFAIP6, PLEK, TNFAIP2, RNF19B, SLC2A3, CXCL1, LITAF, TNF, SAT1, ZFP36, PLAU, ZC3H12A, NAMPT, DENND5A, JUNB, IER2, IER3, MAP2K3, KDM6B, DUSP1, IFNGR2, IL18, PLAUR, TAP1, FOS, RHOB, IL1B, IRF1, NINJ1, TRIB1, SGK1, SQSTM1, CD44, TLR2, B4GALT5, PPP1R15A, CEBPB, PFKFB3, CEBPD, RELA, RELB, SOCS3, SNN, DRAM1, NFIL3, MCL1, STAT5A, EGR1, JUN, GADD45B, LAMB3, TNFRSF9, G0S2, CFLAR, SOD2, PNRC1, KLF2, EIF1, FOSL2, NFKB2, EHD1, PTPRE, MARCKS, BCL6, TNIP1, TNIP2, BCL3, MXD1]
Interferon Gamma Response	4.31E-09	8.11E-08	[IFITM3, OGFR, CDKN1A, IFITM2, EIF4E3, TNFAIP6, TNFAIP2, SECTM1, UBE2L6, ADAR, IFI35, IFI30, IFIT3, OASL, CASP8, NAMPT, CASP4, TNFSF10, PIM1, CASP1, TRIM25, UPP1, TRIM21, ZBP1, IFNAR2, BATF2, IL4R, SP110, HLA-B, TAP1, TRAFD1, HLA-A, HLA-G, TAPBP, PLSCR1, IRF1, IRF2, PSME1, IRF7, SERPING1, LCP2, RBCK1, IRF9, RNF31, C1R, MVP, FPR1, CSF2RB, SRI, PSMB10, HELZ2, SOCS3, HLA-DMA, SLC25A28, FCGR1A, MX2, STAT3, EIF2AK2, ISG15, NMI, TOR1B, SOD2, PSMB8, PML, PSMB9, BST2, ISG20, ZNFX1, PTPN6, MYD88]
Interferon Alpha Response	4.87E-09	8.11E-08	[IFITM3, OGFR, RNF31, IFITM1, IFITM2, UBE2L6, ADAR, IFI35, IFI30, TMEM140, IFIT3, OASL, HELZ2, NUB1, CASP8, SLC25A28, CASP1, MVB12A, TRIM25, GBP2, TRIM21, BATF2, IL4R, SP110, TAP1, TRAFD1, HLA-C, EIF2AK2, ISG15, NMI, PARP9, PSMB8, PSMB9, ISG20, BST2, PLSCR1, SELL, IRF1, IRF2, PSME1, IRF7, IRF9]
Apoptosis	1.02E-08	1.27E-07	[IFITM3, BTG2, CDKN1A, HSPB1, BRCA1, TNF, SAT1, CASP9, CASP8, CASP4, FDXR, TNFSF10, TIMP2, CASP1, TSPO, TIMP1, IER3, BNIP3L, GPX1, GPX4, TNFRSF12A, GPX3, PPP2R5B, IL18, TAP1, EMP1, RHOB, DNAJC3, DDIT3, IL1B, IRF1, RARA, SQSTM1, PLCB2, CD44, RNASEL, DIABLO, NEDD9, BCL10, PSEN1, RELA, PAK1, GNA15, LMNA, CD14, BID, MCL1, BCAP31, JUN, GSN, GADD45B, BIK, CFLAR, SOD2, IGF2R, ISG20, DAP, PPT1, BAX]
IL-6/JAK/STAT3 Signaling	1.94E-08	1.94E-07	[ACVRL1, CSF3R, CXCL1, CSF2RB, IL2RG, CBL, TNF, CSF2RA, PIK3R5, SOCS3, PIM1, BAK1, CD14, CCR1, JUN, IL4R, TGFB1, TNFRSF12A, IL1R1, IL10RB, IL1R2, IFNGR2, STAT3, TYK2, TNFRSF1B, TNFRSF1A, IL17RA, IL1B, IRF1, GRB2, LTB, LTBR, IRF9, CD44, MYD88, TLR2, IFNAR1, PF4]
Inflammatory Response	2.69E-08	2.24E-07	[IFITM1, BTG2, CDKN1A, CSF3R, CD82, TNFAIP6, AQP9, ADM, BEST1, IL18RAP, NAMPT, CLEC5A, TNFSF10, PROK2, FFAR2, TIMP1, SCARF1, SCN1B, IL10, IL4R, IL1R1, IFNGR2, RHOG, IL18, PLAUR, EMP3, TNFRSF1B, TAPBP, TLR1, IL1B, IRF1, IRF7, LTA, LCP2, ITGA5, RAF1, TLR2, IFNAR1, HPN, PTAFR, C5AR1, FPR1, PSEN1, NOD2, SRI, MEFV, RELA, PIK3R5, GNA15, NLRP3, CD14, CD55, LYN, CCL24, GABBR1, SLC31A2, SEMA4D, SLC31A1, TNFRSF9, ADRM1, OSM, EIF2AK2, NMI, BST2, PTPRE, P2RX4, SELL, MXD1]
Complement	6.50E-08	4.64E-07	[FCN1, DGKG, CDA, SERPINA1, ITGAM, CPQ, PLEK, CXCL1, CTSS, PIK3CG, CASP9, CSRP1, LAMP2, CASP4, TIMP2, PIM1, CASP1, TIMP1, CTSD, CTSB, FCER1G, CR1, PRKCD, ANXA5, RHOG, PLAUR, SIRT6, ADRA2B, F5, PLSCR1, IRF1, IRF2, IRF7, SERPING1, LCP2, ANG, PFN1, RAF1, S100A9, CEBPB, C1R, WAS, PRCP, PSEN1, PIK3R5, GNAI2, PPP4C, GNG2, S100A12, CD59, STX4, RCE1, CD55, LYN, GCA, BRPF3, PRSS36, ATOX1, PSMB9, EHD1, GP9, RABIF, GNB2, CALM3, GRB2, HSPA1A, MSRB1]

p53 Pathway	1.78E-06	1.11E-05	[CCNK, BTG2, CDKN1A, CD82, RNF19B, IFI30, SAT1, PITPNC1, FDXR, CASP1, UPP1, CTSD, IER3, PHLDA3, APAF1, ABCC5, DCXR, TAP1, TRAFD1, FOS, PRKAB1, WRAP73, H2AJ, MAPKAPK3, DDIT3, NINJ1, TAX1BP3, S100A4, ISCU, PPP1R15A, CEBPA, NOTCH1, ABHD4, PDGFA, TGFA, XPC, NDRG1, ZFP36L1, SERTAD3, RXRA, DRAM1, IRAK1, RAB40C, MKNK2, SFN, BAK1, S100A10, IP6K2, PLK3, JUN, TGFB1, VDR, OSGIN1, INHBB, BAIAP2, BLCAP, DEF6, H1-2, PTPRE, SP1, BAX, MXD1, RAD9A]
Reactive Oxygen Species Pathway	2.67E-06	1.48E-05	[CDKN2D, G6PD, EGLN2, SRXN1, GPX4, GPX3, NDUFB4, MGST1, LSP1, TXN, SOD2, PRDX6, ATOX1, MSRA, HHEX, SBNO2, FES, NDUFS2, MBP, JUNB, HMOX2, FTL, LAMTOR5]
UV Response Up	7.69E-06	3.85E-05	[BTG2, ALAS1, AMD1, CLTB, ARRB2, SPR, BSG, ACAA1, PRKACA, JUNB, STARD3, DGAT1, GPX3, PRKCD, TAP1, SLC6A12, FOS, HLA-F, TUBA4A, RHOB, CDC34, TST, IRF1, PPIF, TMBIM6, FKBP4, ALDOA, SQSTM1, RPN1, WIZ, FURIN, SULT1A1, RXRB, NXF1, AP2S1, MGAT1, BAK1, BID, YKT6, ATP6V1C1, MARK2, ATP6V1F, LYN, KLHDC3, NTRK3, RAB27A, PDAP1, SOD2, DLG4, PPT1, GRINA]

Supplemental Table S5. Enriched hallmarks in the 2-hours vs Pre and 24-hours vs 2-hours post HTT comparisons. Statistically significant (q-value <0.05) hallmark terms are ordered by q-value, which corresponds to the p-value adjusted for multiple comparisons.
